# Ordinal outcome analysis improves the detection of between-hospital differences in outcome

**DOI:** 10.1186/s12874-020-01185-7

**Published:** 2021-01-06

**Authors:** I. E. Ceyisakar, N. van Leeuwen, Diederik W. J. Dippel, Ewout W. Steyerberg, H. F. Lingsma

**Affiliations:** 1 Centre for Medical Decision Making, Department of Public Health, Erasmus MC - University Medical Center, PO Box 2040, 3000 CA Rotterdam, The Netherlands; 2 Department of Neurology, Stroke Center, Erasmus MC - University Medical Center, Rotterdam, The Netherlands; 3 Department of Medical Statistics and Bioinformatics, Leiden University Medical Center, Leiden, The Netherlands

**Keywords:** Between-hospital variation, Observational data, Comparative effectiveness research, Statistical power, Ordinal outcome analysis, Proportional odds analysis, Benchmarking

## Abstract

**Background:**

There is a growing interest in assessment of the quality of hospital care, based on outcome measures. Many quality of care comparisons rely on binary outcomes, for example mortality rates. Due to low numbers, the observed differences in outcome are partly subject to chance. We aimed to quantify the gain in efficiency by ordinal instead of binary outcome analyses for hospital comparisons. We analyzed patients with traumatic brain injury (TBI) and stroke as examples.

**Methods:**

We sampled patients from two trials. We simulated ordinal and dichotomous outcomes based on the modified Rankin Scale (stroke) and Glasgow Outcome Scale (TBI) in scenarios with and without true differences between hospitals in outcome. The potential efficiency gain of ordinal outcomes, analyzed with ordinal logistic regression, compared to dichotomous outcomes, analyzed with binary logistic regression was expressed as the possible reduction in sample size while keeping the same statistical power to detect outliers.

**Results:**

In the IMPACT study (9578 patients in 265 hospitals, mean number of patients per hospital = 36), the analysis of the ordinal scale rather than the dichotomized scale (‘unfavorable outcome’), allowed for up to 32% less patients in the analysis without a loss of power. In the PRACTISE trial (1657 patients in 12 hospitals, mean number of patients per hospital = 138), ordinal analysis allowed for 13% less patients. Compared to mortality, ordinal outcome analyses allowed for up to 37 to 63% less patients.

**Conclusions:**

Ordinal analyses provide the statistical power of substantially larger studies which have been analyzed with dichotomization of endpoints. We advise to exploit ordinal outcome measures for hospital comparisons, in order to increase efficiency in quality of care measurements.

**Trial registration:**

We do not report the results of a health care intervention.

**Supplementary Information:**

The online version contains supplementary material available at 10.1186/s12874-020-01185-7.

## Background

There is an ever-growing demand for information on performance of hospitals to improve quality of care [1]. Clinical outcomes are commonly used to determine which hospitals are allegedly performing better or worse, and which are to be labelled as potential outliers [2, 3]. However, comparing outcomes between hospitals has its limitations. The observed differences in outcome between hospitals are often partly due to chance [4] and are only partly explained by actual differences in the quality of care [5]. Lack of power to detect differences between hospitals is a common problem for several clinically relevant outcome indicators. For example, complication rates are generally low and the small number of events leads to underpowered statistical analyses [6, 7]. Furthermore one of the most commonly used clinical measures is the (standardized) mortality ratio (SMR) [8, 9], which has a variety of disadvantages and methodological issues when used as a quality of care measure [10–12]. The main issue being that mortality is an especially rare outcome in many patient groups, leading to low power when trying to detect hospitals with aberrant outcomes [13].

Many clinical continuous or ordinal outcome scales do exist and are recorded, but these are often dichotomized (favorable and unfavorable) in quality of care comparisons, for reasons of simplicity. Examples of ordinal outcome measures are the modified Rankin Scale (for stroke), the Glasgow Outcome Scale (for Traumatic Brain Injury (TBI), the Guillain Barré syndrome disability score, the NYHA Functional Classification (for heart failure) and the Rutherford Classification (for peripheral artery disease). Dichotomization has been shown to lead to a loss of clinically and statistically relevant information in several studies [14–17] while analysis on the full ordinal scale with proportional odds analysis, prevents this loss of information [18, 19]. Simulation studies and empirical validation studies in clinical trials have demonstrated that ordinal analysis increases statistical power compared to binary outcome analysis [18–21]. For clinical trials it has already been advised not to dichotomize ordinal outcome scale but to exploit the full ordinal nature of the scale, to allow for detection of smaller treatment effects [15, 19]. However, this potential gain in efficiency has not been assessed for hospital comparisons.

Our aim, therefore, is to quantify the gain in power, or reduction in sample size, that can be achieved by using ordinal compared to dichotomous outcomes as a measure of quality of care for hospital comparisons.

## Methods

Simulation studies were performed with patients sampled from two databases. The databases consisted of hospital data of patients with either TBI in the International Mission on Prognosis And Clinical Trial Design in Traumatic Brain Injury (IMPACT) study [22], and stroke patients in the PRomoting ACute Thrombolysis in Ischemic StrokE (PRACTISE) trial [23].

The IMPACT study was a project was organized as a collaborative venture between the Erasmus University in Rotterdam, The Netherlands, the University of Edinburgh, Scotland, and the Virginia Commonwealth University Medical College in Richmond, Virginia in order to collect data from available randomized controlled trials (RCTs) and observational studies in TBI conducted between 1984 and 2007. Although inclusion criteria differed for the different RCTs data extraction was guided by a data dictionary to standardize the format of variables entered into data set, to guarantee the quality of the data.

The PRACTISE study is a national cluster-randomised-controlled trial. All patients > 18 years with acute stroke who were admitted to the hospital within 24 h from onset of symptoms were included in the trial. Patients admitted within 4 h were assessed in detail and were followed up to 3 months after onset by telephone. Twelve hospitals participating hospitals were assigned to the regular or high-intensity intervention.

**Fig. 1:**
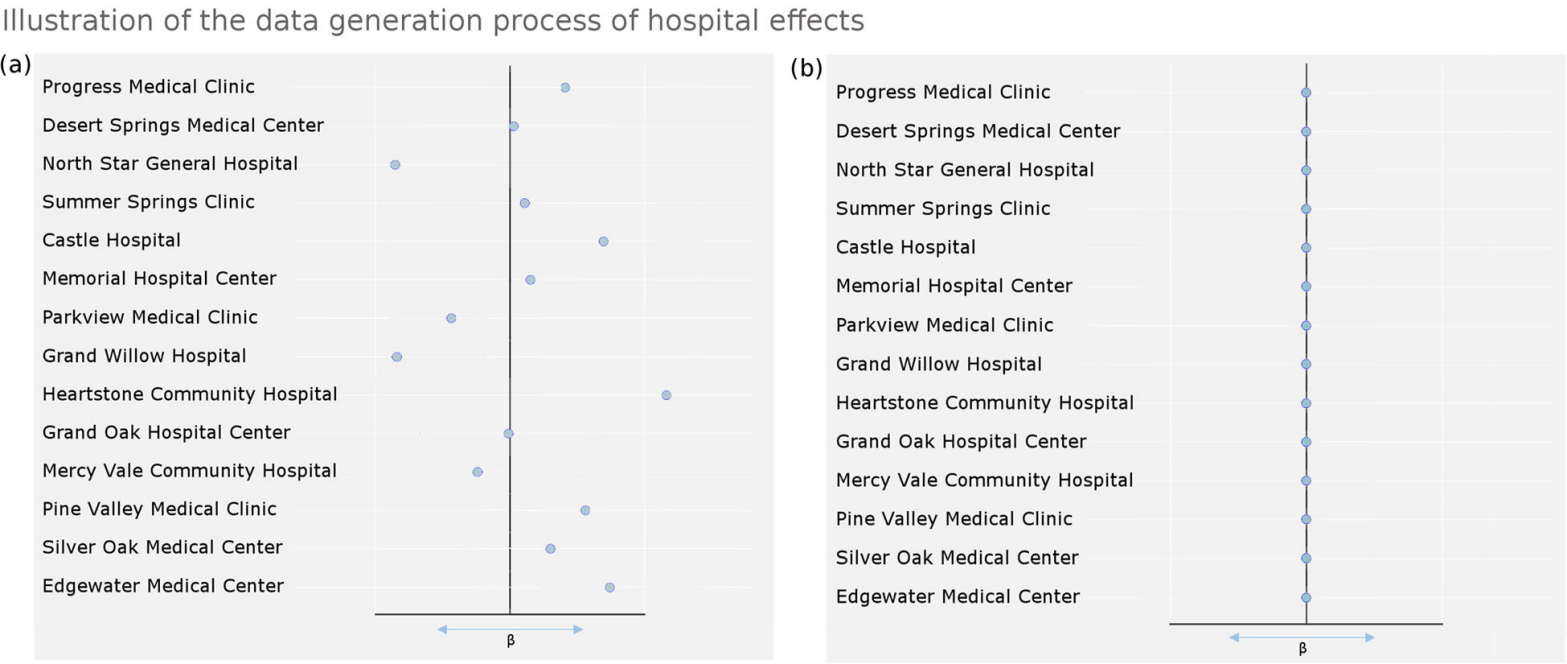
Illustration of the data generation process for testing specificity and sensitivity: (**a**) when a center effect is added (β), resulting in every hospital performing to different degrees, better or worse than the mean (**b**) without a hospital effect added (β = 0) all hospitals perform the same

We sampled patients from the original data sets and the patients were appointed to one of the 250 fictitious hospitals. The simulations included two scenarios, one in which the hospital influenced the outcome of the patient (A) and one in which outcome of the patient was completely independent of the hospital (B). In the first scenario (A) hospitals were given a “center effect”; a coefficient for the effect of hospital on outcome drawn from a normal distribution with m = 0 and SD = 0.35. The true outcomes of the hospitals all differed from 0, as can be seen in Fig. 1a. This meant that patients from one hospital had a higher chance of a good outcome than those of another [20], i.e. that ‘true’ hospital differences in outcome existed. Analysis of scenario A was done to determine the sensitivity (type II error) which could be achieved using either ordinal outcomes or dichotomized outcomes. Since all hospitals were assigned a center effect, the analysis which found the most hospitals with performances deviating from the mean had the best sensitivity for an effect.

In scenario B specificity was tested (Fig. 1b) by checking if the analyses did not find more than 5% of differently performing hospitals when there was no true difference in hospital performance.

To simulate outcomes, a multinomial generalized logit regression model was fitted to predict the probability for outcomes for each patient based on the given baseline covariates. Furthermore, in scenario *A* the probability for outcome was either increased or decreased depending on the hospital the patient was in.

For the baseline covariates of the TBI patients well know prognostics baseline characteristics were used: Glasgow Coma Scale (GCS) motor score, age and pupillary reactivity (both pupils reactive, one pupil reactive, no pupil reactivity) [22]. In the stroke data the following covariates were used: baseline National Institute of Health Stroke Scale (NIHSS) score, age, history of ischemic stroke, atrial fibrillation, an diabetes mellitus [23].

**Fig. 2:**
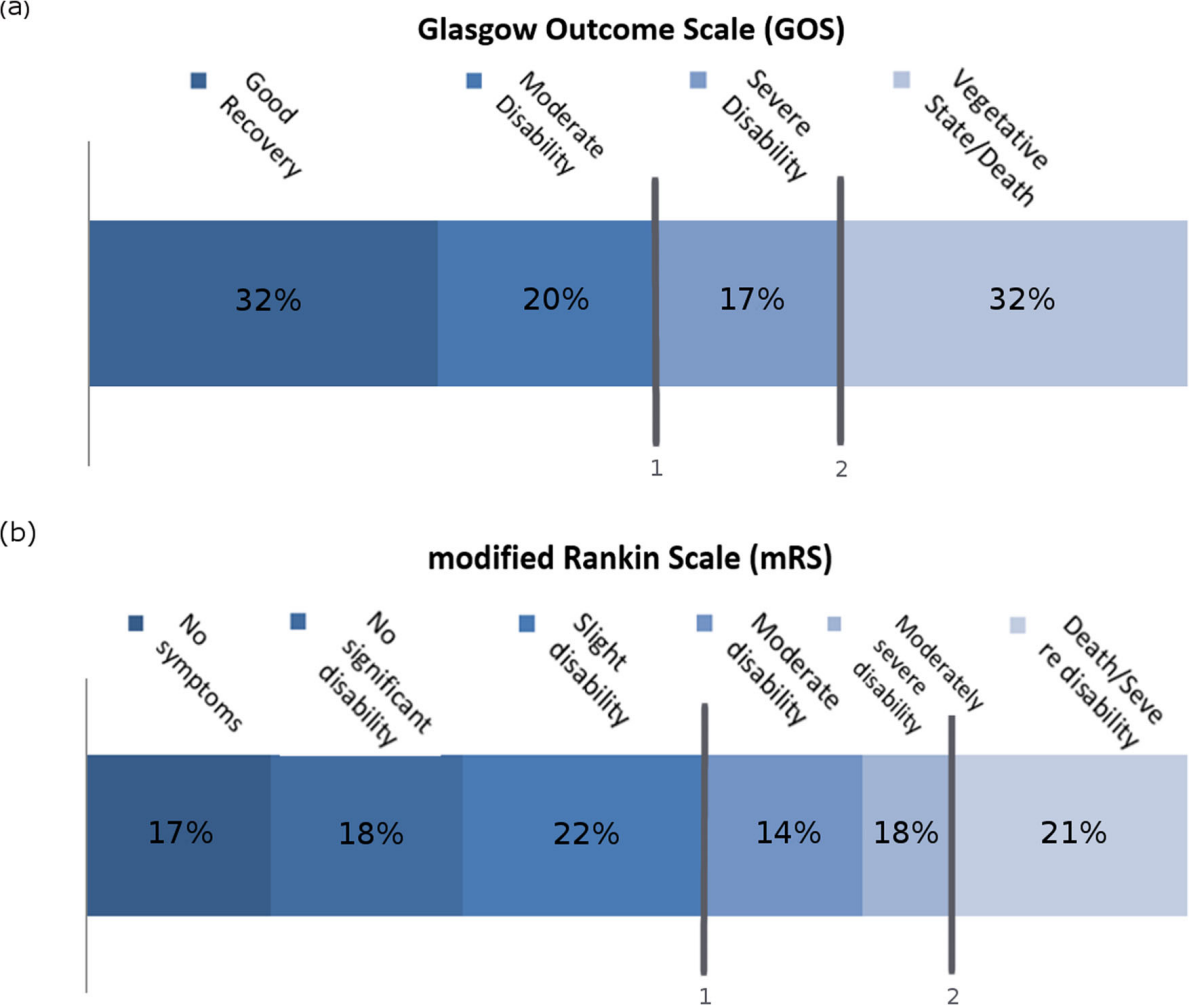
Distributions of the Glasgow Outcome Scale (**a**) and the modified Rankin scale (**b**), with the vertical line **1** illustrating the point of dichotomization at the clinically relevant outcome, and line **2** illustrating the point of dichotomization for mortality

In TBI, we used the 5-point ordinal Glasgow Outcome Scale (GOS) at 6-months as an outcome measure (Fig. 2a). For stroke, we used the modified Rankin Scale (mRS) at 3 months, a 7-point ordinal scale (Fig. 2b). These are the most commonly used outcome measures for these conditions. In both scales, the worst disability state and death were combined for ethical reasons, resulting in a 4-point outcome scale for TBI, and a 6-point outcome scale for stroke [19, 23, 24]. Both ordinal outcome measures were thereafter dichotomized, into favorable (good recovery or moderate disability) and unfavorable outcome (severe disability, vegetative state and death) as well as dichotomized for mortality (including severe disability). Dichotomization for mortality was done to illustrate the case in which only mortality rates are measured.

To demonstrate the differences in sensitivity to detect hospital outliers the simulation was repeated with different number of patients per hospital, ranging from 25 to 200, which were distributed over 250 hospitals. Simulations were run 500 times.

### Analysis

Outcomes were analyzed on (1) an ordinal scale, (2) dichotomized as favorable vs. unfavorable outcomes, and (3) dichotomization for death vs. alive. The binary outcomes were analyzed with standard fixed effect logistic regression models, the ordinal outcomes were analyzed with proportional odds fixed effect logistic regression models [25, 26]. All models were adjusted for previously mentioned baseline covariates based on which the outcomes had been predicted, and included hospital as a categorical variable. This yielded an estimated center effect per hospital compared to mean center effects. Hospitals with predicted center effect values outside the 95% confidence intervals (CIs) of the overall mean were scored as outliers.

The ability of the model to determine which hospitals were outliers was measured by counting how many outliers the analysis would find in different scenarios. This means that in scenario A, the analysis which found the most outliers was determined as the most sensitive, and in scenario B the analyses were meant to have less than 5% outliers.

Higher power in the analyses results in higher rate of correctly identified outliers. Therefore, we could translate the ability to find outliers to the possibility of sample size reduction. The ability of regression models to determine which hospitals had aberrant outcomes, given dichotomized and ordinal outcomes was expressed in potential efficiency gains. The difference between ordinal and dichotomized outcomes was expressed as potential efficiency gain: the possible reduction in sample size while keeping the same statistical power to detect outliers. All analyses were done using R Statistical Software 3.3.0. The script can be found in Appendix [27–33].

## Results

The IMPACT study included data from eight randomized controlled trials and three observational studies [22]. Data from 9578 patients was used, which came from 265 different centers, which admitted between 1 and 453 patients, which were mostly (78%) male, and had a median age of 30 (interquartile range (IQR): 21–45) (Table 1).

**Table 1:** Baseline characteristics of patients enrolled in the IMPACT study

	***n*** = 9578
Age (median, IQR)	30 (21–45)
Sex	
Male (N, %)	7446 (78%)
Pupillary reactivity	
Reactive to light (N, %)	7664 (80%)
Not reactive to light (N, %)	1914 (20%)
Motor score	
Makes no movements (N, %)	1490 (16%)
Extension to painful stimuli (N, %)	1166 (12%)
Abnormal flexion to painful stimuli (N, %)	1244 (13%)
Flexion / Withdrawal to painful stimuli (N, %)	2208 (23%)
Localizes to painful stimuli (N, %)	2593 (27%)
Obeys commands (N, %)	291 (3%)

The PRACTISE trial was a cluster randomized trial of studying the implementation of IV thrombolytic treatment in the Netherlands. It included observational data of 1657 patients in 12 centers [23]. Hospitals had a minimum of 28 and maximum of 310 patients, who had a median age of 73 (IQR: 62–80) (Table 2).

**Table 2:** Baseline characteristics of patients enrolled in the PRACTICE trial

	***n*** = 1657
Age (median, IQR)	73 (62–80)
Sex	
Male (N, %)	902 (54%)
Atrial fibrillation	
Present (N, %)	296 (18%)
Not present (N, %)	1361 (82%)
Diabetes mellitus	
Present (N, %)	274 (17%)
Not present (N, %)	1383 (84%)
History of ischemic stroke	
Present (N, %)	331 (20%)
Not present (N, %)	1326 (80%)
NIHSS^a^(mean)	8

^a^NIHSS indicates National Institutes of Health Stroke Scale; indicator of Stroke severity

In the IMPACT study 4949 (52%) of the patients had a favorable outcome and 4629 (48%) had an unfavorable outcome. Of these, 3031 (32%) were in vegetative state or died (Fig. 2). In the PRACTISE trial 933 (56%) of the patients had a favorable outcome and 724 (44%) had an unfavorable outcome. Of these, 351 (21%) were in severely disabled state or died.

### Sensitivity

**Fig. 3:**
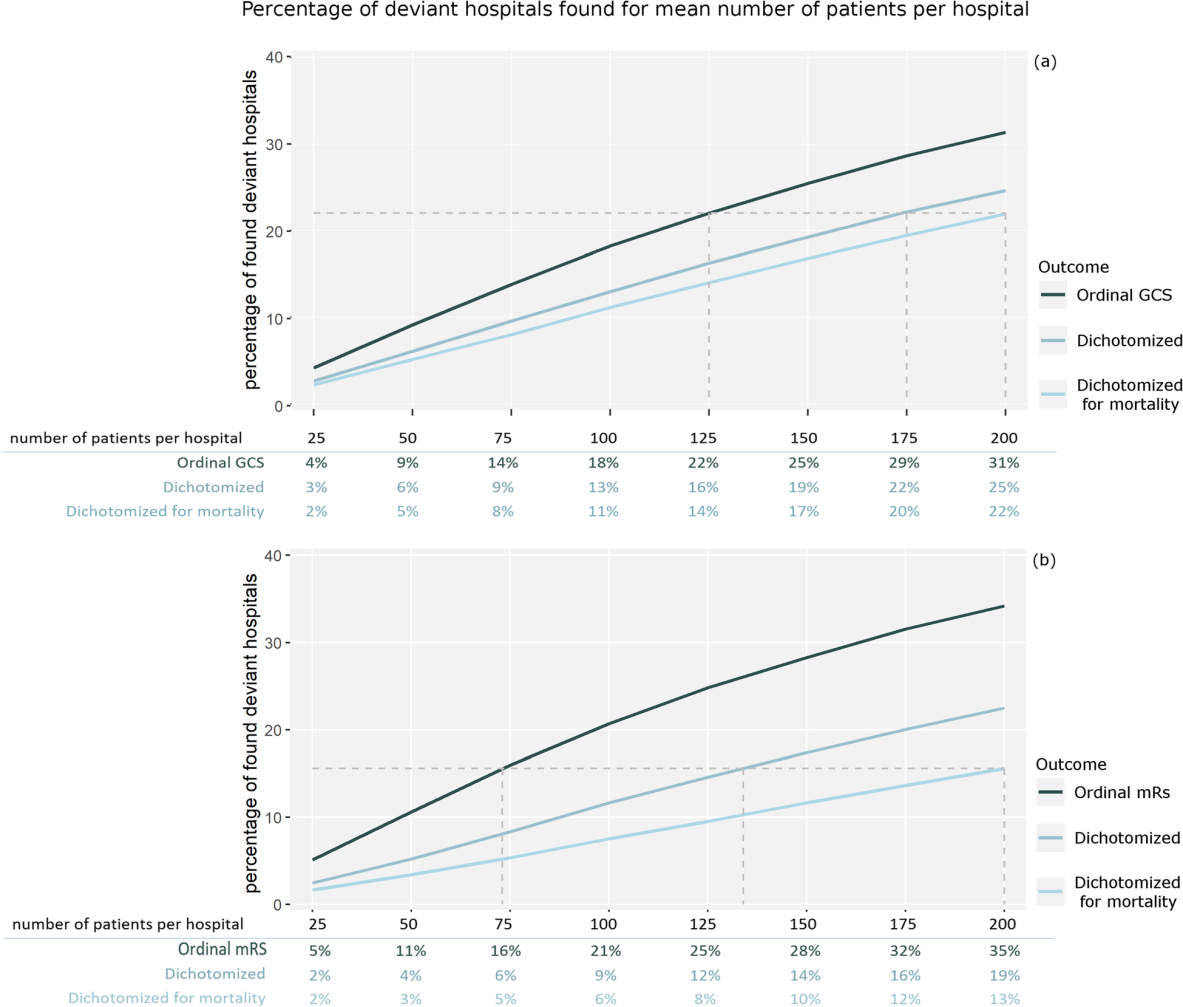
Results of the simulation based on the IMPACT database (**a**) and results of the simulation based on the PRACTICE trial (**b**). The graph shows mean number of patients which need to be included per hospital in order to be able to find the number better or worse performing hospitals, set out for data which has been dichotomized, dichotomized for mortality/severe disability, and which was analyzed respectively on the full ordinal GOS scale (**a**), the modified Rankin scale (**b**)

More patients per hospital increased the percentage of hospitals which are correctly found to be deviant from the mean (Fig. 3). Further, use of ordinal outcomes instead of dichotomized for favorable versus unfavorable outcome allowed for less patients in the analysis without loss of power; the use of ordinal outcomes compared to dichotomized outcomes allowed for up to 13% less patients in the analysis without a loss of power in the IMPACT study (Fig. 3a) and for up to 32% less patients in the PRACTISE trial (Fig. 3b). For example, a mean of 73 patients per hospital was needed to detect the same percentage deviant hospitals when ordinal outcomes were used compared to on average 134 patients per hospital when the dichotomization favorable versus unfavorable was used in the PRACTISE trial. Moreover, dichotomization for mortality required even more patients in the analysis compared to dichotomization for favorable versus unfavorable outcome, in this example 200 patients per hospital. This meant that the required number of patients could be reduced by 63% for the PRACTISE trial and up to 37% for the IMPACT study. The variation across simulations was relatively small (Appendix Figure 4).

### Specificity

To determine specificity, the simulations were performed without simulating true center effects. For all analyses an increase in sensitivity was not associated with a decrease in specificity: the type I error did not differ between analytical approached and was in all cases below 1%.

## Discussion

This study aimed to assess how much power could be gained by using ordinal analysis instead of dichotomous analysis to detect between center differences in outcome. Use of ordinal outcomes in both stroke and TBI hospital comparisons, increased statistical efficiency of the estimation of differences between centers. The increase in statistical power resulted in a substantial reduction in required sample size when using ordinal instead of dichotomous outcomes. This sensitivity increase came without loss of specificity.

Our results are in line with previous studies on estimating treatment effects in RCTs [34–36]. Previous studies on ordinal outcome analysis in trials, showed an increase in power, and higher potential of detecting treatment effects [15, 19, 20, 37], with sample size reductions up to 40%. The current study shows the use of ordinal data is not only of added value in RCTs that assess treatment effects, but also in observational data to assess differences between centers in outcome. It illustrates to what extent sample size can be reduced without loss of power compared to the use of a dichotomous outcome. In the example databases on stroke and TBI, a reduction in sample size of 37 and 63% was achieved. The difference in power gain between the two examples could be partly explained by the fact that the mRS is used as a 6-point ordinal scale (originally 7) while the GOS is used as a 4-point ordinal scale (originally 5). An ordinal scale with a higher number of levels may contain more information, and may provide more discriminability. In addition, the efficiency gain of an ordinal outcome is optimal if the proportional odds assumption perfectly holds [38, 39].

In our analysis we used odds based on the true data from the IMPACT study and PRACTICE trial, in which the proportional odds assumption is not perfectly met. It has however been shown that even if proportional odds assumptions are violated, analysis of the ordinal scale is still beneficial over dichotomization and results are robust regardless of the violation [20, 40, 41]. In the past the importance of assumption of proportionality might have been stressed too much. More important than the proportional odds assumption is the ordering of the adjacent outcomes. If there is agreement among stakeholders that each score on a certain scale is more favorable than a one point lower score, testing for proportional odds assumptions can be considered redundant [41]. If not, a potential solution is to combine adjacent categories of the scale that are not perceived ordinal, e.g. dead and vegetative state.

This study illustrates how much information is lost, not only by discarding the ordinal outcomes but when dichotomization leads to low event rates. This is the case when only mortality ratios are considered, and especially when mortality at a fixed time point is used. Compared to ordinal outcomes mortality as outcome requires much larger sample sizes, in order to find potential differences in quality of care.

Using ordinal outcomes, when available, instead of dichotomous outcomes to compare hospitals is therefore strongly recommended. For stroke and TBI this is easily done, as most centers will be familiar with the use of these scales in research projects and clinical practice. We do however recognize that several medical conditions or fields do not have a relevant ordinal outcome scales. Ideally relevant ordinal scales for important conditions should be developed or refurbished and implemented.

The benefits of the use of ordinal scales have also been shown to have their limits. The chance of misclassification, even by extensively trained medical staff, is higher with the use of ordinal scales. This phenomenon is represented as the inter-rater reliability [42]. Misclassification has been included in the simulation, if misclassification is however larger than expected it can possibly lead to an underestimation of the error rates and an overestimation of the statistical power of the ordinal analyses [42–46]. Furthermore, in our analysis we collapsed vegetative state and mortality for the GOS, and similarly we collapsed mRS 5 (severe disability) with mRS 6 (death) into one state. For the GOS it is more of a common practice since it is questionable whether vegetative state is a better outcome than mortality. This has also been done for mRS, although patients in mRS 5 are awake and aware, and on average this is clearly a preferred health status over death. Clinically this might be a debatable choice, it is however done on occasion and in our analysis it makes comparison to the GOS easier and yields a more conservative estimate of the gain in power [23, 24].

Dichotomization was done on the collapsed scale which adds possible misclassification to the dichotomized outcome scale, while true mortality ratios would not have any misclassification. At the same time including vegetative state and severe disability cases increases incidence rates and therefore the power of the analysis.

In this paper we repeatedly refer to reduction in sample size using ordinal instead of dichotomous outcomes. However, since statistical power is a major challenge in hospital comparisons, we would like to stress that by this we point out efficiency gain by using ordinal outcome analysis. Most (studies on) hospital comparisons are underpowered, and thus we do not advise aiming for smaller sample sizes when using ordinal outcomes. In this paper only one aspect in performing hospital comparisons is addressed. In general, to be able to perform valid and efficient hospital comparisons one should focus on 1) using larger sample sizes [47], 2) use ordinal outcome analyses and 3) sufficient case-mix adjustment.

### Strengths and limitations

The advantage of performing simulations on quality of care data is that we have a priori knowledge of which hospitals deviate from the mean. A limitation of basing the simulation on real datasets is that it limits the variety of situations which are simulated. Furthermore, the number of patients per hospital was constant in our study, instead of a mix of smaller and larger hospitals as one would see in reality.

## Conclusion

Use of the ordinal outcomes instead of the binary outcomes for hospital comparisons, results in considerable efficiency gains. In quality of care research, where lack of power is a substantial problem, using ordinal clinical outcomes could be a way to increase possibilities to find outliers when comparing hospitals. In cases where an ordinal scale is available we strongly advise to exploit the ordinal scale and to not dichotomize in any way.

### Supplementary Information

**Additional file 1.**

## Figures and Tables

**Fig. 4:**
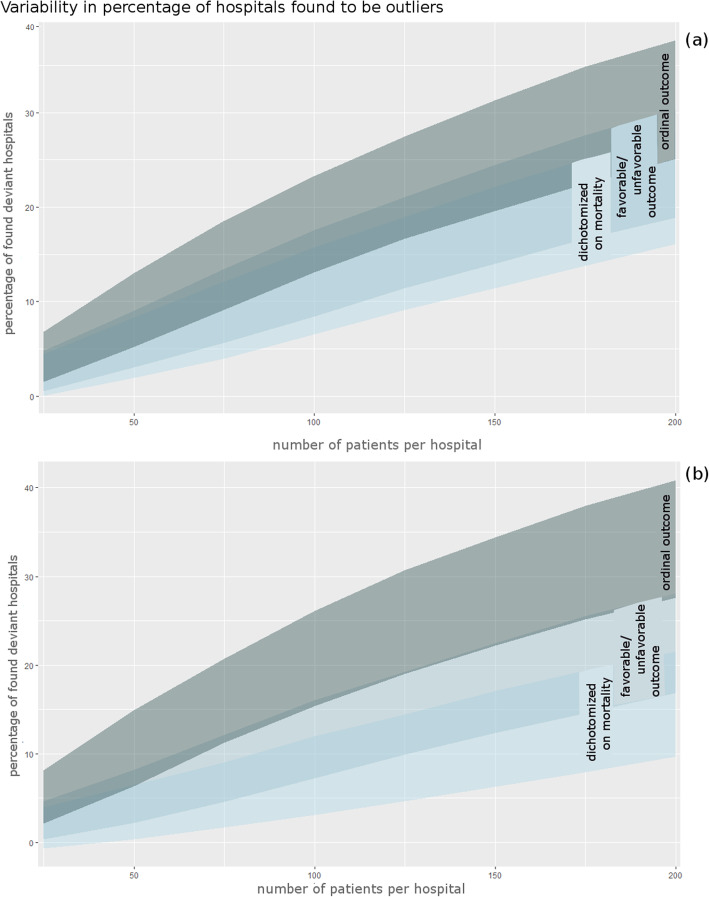
Results of the simulation based on the IMPACT database (**a**) and results of the simulation based on the PRACTICE trial (**b**). The graph shows the variability in number of patients which need to be included per hospital in order to be able to find the number better or worse performing hospitals, set out for data which has been dichotomized, dichotomized for mortality/severe disability, and which was analyzed respectively on the full ordinal GOS scale (**a**), the modified Rankin scale(**b**)

**Fig. 5:**
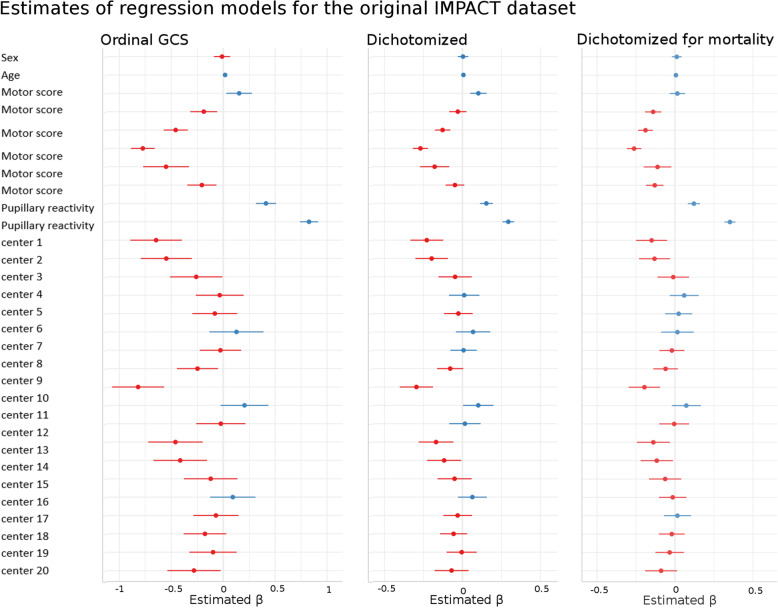
Estimates from the regression models based on the original IMPACT database for data which has been analyzed on the full ordinal GOS scale, GOS dichotomized, and GOS dichotomized for mortality/severe disability

## Data Availability

The datasets generated and/or analysed during the current study are not publicly available because participants gave no consent for data sharing.

## Abbreviations

TBI: Traumatic brain injury
SMR: Standardized mortality ratio
IMPACT: International Mission on Prognosis And Clinical Trial Design in Traumatic Brain Injury
PRACTISE: PRomoting ACute Thrombolysis in Ischemic StrokE
GCS: Glasgow Coma Scale
mRS: Modified Rankin Scale
NIHSS: National Institute of Health Stroke Scale
CIs: Confidence intervals
IQR: Interquartile range
