# Supplementary material for: Ordinal outcome analysis improves the detection of between-hospital differences in outcome
Source: BMC Med Res Methodol. 2021 Jan 6;21:4. doi: 10.1186/s12874-020-01185-7 (PMC7788719; doi:10.1186/s12874-020-01185-7)
Supplement: Supplementary file 1 — Additional file 1. [file 12874_2020_1185_MOESM1_ESM.docx]

R-code

########## LIBRARIES #########

library(Hmisc)

library(rms)

library(foreign)

library(lme4)

library(gtools)

library(mice)

library(mlogit)

library(VGAM)

library(memisc)

######### SET PARAMETERS ###########

#minimum patients per centre

minimum_pat <- 10

# nAGQ

value_nAGQ <- 15

#Z-value

z_value <- 1.96

#set.seed(1)

options(scipen = 20)

# vector of mean patients per hospital

n.vec = seq(25,200, by = 25)

# xfold

xfold = 4

# repeat simulation n times

reps = 500

######### ANALYSIS ###########

# LOAD DATA

tbi.data <- read.spss('../tbi_data.sav', to.data.frame = TRUE, use.value.labels = FALSE)

# FILTER

# subset only with hospitals with more than 10 patients

number_patients<-group_by(tbi.data, center) %>%

summarise(count = n())

filt.data <- merge(tbi.data, number_patients, by= "center", all.x = TRUE)

filt.data<-subset(filt.data, filt.data$count>=10)

######### SIMULATION ###########

#new data frame for simulated outcomes

sim.data <- data.frame(...)

# make a data frame with "sample_size" patients, randomly drawn from data

filt.data <- filt.data[sample(1:nrow(filt.data),size = sample_size, replace=TRUE),]

# give new names to avoid duplicate row names

row.names(filt.data) <- c(1:sample_size)

#create right data type for mlogit

mlogit.data <- mlogit.data(o_filtered, shape= "wide", choice = "d_gos", alt.levels = c(1, 2, 3, 4, 5))

#fit a pure "multinomial model"

fit <- mlogit(d_gos ~ 0 |age + d_motor + trial + i_pupil, data = mlogit.data)

## calculae chances of outcome for each patient

# matrix with the chances of each outcome for each patient

chance_dgos <- fit$probabilities

# make new outcomes: draw randomly from multinomial with predicted chances

sim.data$d_gos <- rMultinom(chance_dgos,1)

#calcultae odds ratios

OR4 = chance_dgos[,4]/(1-chance_dgos[,4])

OR34 = (chance_dgos[,3] + chance_dgos[,4]) /(1-chance_dgos[,3] - chance_dgos[,4])

OR234 = (chance_dgos[,2] + chance_dgos[,3] + chance_dgos[,4])/(1-chance_dgos[,2] - chance_dgos[,3] - chance_dgos[,4])

## simulating the new centers

#take number of patients

sumcenter <- rep(n.vec[length(n.vec)],250)

# cummulated sum of the patients per row

accum <- cumsum(sumcenter)

# l is number of centers to get near the sample size

l = length(accum[accum<=sample_size])

# the new hospital names

cnames <- c(1:l)

# vector of times to repeat each center name/numbers of patient per center

times <- as.vector(sumcenter)[0:l]

times[length(times)] <- times[length(times)] + (sample_size- accum[length(accum[accum<=sample_size])])

# repeat the center names as many times as needed

# fill with the new center names

sim.data$cnames <- sample(rep(cnames,times), size = sample_size, replace = FALSE)

# matric of centers, dummy varaible

centermatrix <- dummy(rep_cnames, levelsToKeep=c(1:l))

## set new pat id

sim.data$patid <-c(1:nrow(sim.data))

kmatrix <<- matrix(nrow = length(cnames), ncol = 1)

## set new outcomes adding center effects

# draw from normal distribution with SD of log(xfold)/3.92

randeff <- rnorm(l,0,log(xfold)/3.92)

k <- (centermatrix %*% randeff)

kmatrix <<- randeff

# calculate chances

cc_dgos = chance_dgos

cc_dgos[,4] = 1/(1+1/(OR4*exp(k)))

cc_dgos[,3] = 1/(1+1/(OR34*exp(k)))- cc_dgos[,4]

cc_dgos[,2] = 1/(1+1/(OR234*exp(k)))- cc_dgos[,3] - cc_dgos[,4]

cc_dgos[,1] = 1 - cc_dgos[,2] - cc_dgos[,3] - cc_dgos[,4]

# calculate d_gos

sim.data$d_gos <- rMultinom(cc_dgos,1)

# new values for d_unfav

sim.data[sim.data$d_gos==3 | sim.data$d_gos==4,]$d_unfav <- 1

sim.data[sim.data$d_gos==1 | sim.data$d_gos==2,]$d_unfav <- 0

# new values for SMR

sim.data[sim.data[,"d_gos"] < 4,"SMR"] <- 0

sim.data[sim.data[,"d_gos"] > 3,"SMR"] <- 1

# For every N, fit every regression model, and return a list of data frames with the outliers

# (name of the center)

for(i in 1:length(sim.data)){

d = datadist(sim.data)

##### FIXED EFFECTS models #####

adjusted.smr <- (SMR ~ centername + age + d_motor + i_pupil)

adjusted.dich <- (d_unfav ~ centername + age + d_motor + i_pupil)

adjusted.ord <- (d_gos ~ centername + age + d_motor + i_pupil)

FEfit<- lrm(adjusted.ord, data=sim.data)

FEfit<- lrm(adjusted.dich, data=sim.data)

FEfit<- lrm(adjusted.smr, data=sim.data)

#get coefficients of the centres from the fit

fcoef <- as.numeric(FEfit$coefficients[cent_start:center_count])

#add 0 to the first line, instead of intercept

fcoef[1] <- 0

# subtract mean to scale around mean instead of first

fcoef <- fcoef - mean(fcoef)

sd <- sqrt(diag(vcov(fitf)))

results<- as.vector(fcoef)

#confidence intervals

ci.results <- results + 1.96 * outer(sd[cent_start:center_count],

c(lower=-1, upper=1))

# calculate outliers

outliers_ord <- analysis[analysis[,"UPPER_CI"] < 0 | analysis[,"LOWER_CI"] > 0,1]

outliers_dich <- analysis[analysis[,"UPPER_CI"] < 0 | analysis[,"LOWER_CI"] > 0,1]

outliers_smr <- analysis[analysis[,"UPPER_CI"] < 0 | analysis[,"LOWER_CI"] > 0,1]

}
